# Supplementary material for: Effect of Previous Crops and Soil Physicochemical Properties on the Population of Verticillium dahliae in the Iberian Peninsula
Source: J Fungi (Basel). 2022 Sep 21;8(10):988. doi: 10.3390/jof8100988 (PMC9605609; doi:10.3390/jof8100988)
Supplement: Supplementary file 1 [file jof-08-00988-s001.zip › jof-1920161-supplementary.pdf]

Journal of Fungi

MDPI

Supplementary Table S1. Attributes of fields sampled for *Verticillium dahliae* microsclerotia in the Iberian Peninsula.

City	District	Coutry	Field	Field number	Latit.	Longit.	Soil texture	Previous cropping history	Ms/g soil
Alcolea del Río	Cordoba	Spain	Jose Ramirez (Cerca Casa)	**1**	-4.6719	37.9327	Clay	Barely favourable	0
Alcolea del Río	Cordoba	Spain	Jose Ramirez (Pendiente)	**2**	-4.6719	37.9327	Clay loam	Barely favourable	0
Avis	Portalegre	Portugal	Neto Valente (Avis)	**3**	-7.8912	39.0569	Sandy clay loam	Barely favourable	0.8
Belalcázar	Cordoba	Spain	Francisco Ollero	**4**	-5.1561	38.5776	Loam	Barely favourable	0
Elvas	Portalegre	Portugal	Francisco Borba	**5**	-7.1636	38.8801	Loam	Barely favourable	0.4
Elvas	Portalegre	Portugal	Courela do lobo (Elvas)	**6**	-7.1636	38.8801	Clay loam	Barely favourable	0
Elvas	Portalegre	Portugal	Boavista(Elvas)	**7**	-7.1636	38.8801	Clay loam	Barely favourable	0
Ferreira do Alentejo	Beja	Portugal	Jordi Salvado (Carretera)	**8**	-8.1037	38.0578	Sandy clay loam	Barely favourable	0
Ferreira do Alentejo	Beja	Portugal	Jordi Salvado(Balsa)	**9**	-8.1037	38.0578	Sandy clay loam	Barely favourable	0
Ferreira do Alentejo	Beja	Portugal	Jordi Salvado M1	**10**	-8.1037	38.0578	Sandy clay loam	Barely favourable	0
Ferreira do Alentejo	Beja	Portugal	Jordi Salvado M2	**11**	-8.1037	38.0578	Sandy clay loam	Barely favourable	0
Ferreira do Alentejo	Beja	Portugal	Jordi Salvado M3	**12**	-8.1037	38.0578	Sandy clay loam	Barely favourable	0
Ferreira do Alentejo	Beja	Portugal	Jordi Salvado M4	**13**	-8.1037	38.0578	Sandy loam	Barely favourable	0
Ferreira do Alentejo	Beja	Portugal	Jordi Salvado M5	**14**	-8.1037	38.0578	Sandy clay loam	Barely favourable	0

(Continued)

Supplementary Table S1. Continued.

City	District	Coutry	Field	Field number	Latit.	Longit.	Soil texture	Previous cropping history	Ms/g soil
Ferreira do Alentejo	Beja	Portugal	Jordi Salvado M6	**15**	-8.1037	38.0578	Sandy loam	Barely favourable	0
Ferreira do Alentejo	Beja	Portugal	Ferreira do Alentejo(Savo) Camino del pino izquierda	**16**	-8.1037	38.0578	Sandy clay loam	Barely favourable	0
La Carlota	Cordoba	Spain	Federico Garcia M1	**17**	-4.9338	37.6726	Sandy loam	Barely favourable	0
Macossa	Lisbon	Portugal	Casa Agricola Antunes Barroso M1	**18**	-8.8638	39.1954	Clay loam	Barely favourable	0.4
Macossa	Lisbon	Portugal	Casa Agricola Antunes Barroso M2	**19**	-8.8638	39.1954	Loam	Barely favourable	0
Macossa	Lisbon	Portugal	Casa Agricola Antunes Barroso M3	**20**	-8.8638	39.1954	Clay loam	Barely favourable	0
Macossa	Lisbon	Portugal	Casal das Sarnadas S.A.	**21**	-8.8638	39.1954	Sandy loam	Barely favourable	0
Medina del Campo	Valladolid	Spain	Miguel Angel Buitrago (Parcela Grande)	**22**	-4.915	41.3084	Sandy clay loam	Barely favourable	0
Montoro	Cordoba	Spain	El Cerote (Jose Fernando Aguilar)	**23**	-4.3835	38.0227	Clay	Barely favourable	0
Olivenza	Badajoz	Spain	Valondo (Delantera)	**24**	-7.1026	38.6826	Sandy clay loam	Barely favourable	0
Olivenza	Badajoz	Spain	El Cedeño (Franca)	**25**	-7.1026	38.6826	Sandy clay loam	Barely favourable	0
Olivenza	Badajoz	Spain	Valondo (Trasera)	**26**	-7.1026	38.6826	Sandy loam	Barely favourable	0
Palma del Río	Cordoba	Spain	Antonio Valle (Suelo negro)	**27**	-5.2802	37.6982	Clay	Barely favourable	2.4
Palma del Río	Cordoba	Spain	Antonio Valle (Suelo chinarro)	**28**	-5.2802	37.6982	Clay	Barely favourable	1.2
Portillo	Valladolid	Spain	Victor Fernandez 1	**29**	-4.5728	41.4806	Sandy	Barely favourable	0
Portillo	Valladolid	Spain	Victor Fernandez 2	**30**	-4.5728	41.4806	Sandy	Barely favourable	0
Santaella	Cordoba	Spain	Federico Garcia M2	**31**	-4.843	37.5622	Clay	Barely favourable	1.2
Santaella	Cordoba	Spain	Federico Garcia M4	**32**	-4.843	37.5622	Clay	Barely favourable	0

(Continued)

Supplementary Table S1. Continued.

City	District	Coutry	Field	Field number	Latit.	Longit.	Soil texture	Previous cropping history	Ms/g soil
Tocina	Seville	Spain	Jose Antonio Espinar (Parcela Grande)	**33**	-5.7339	37.61	Sandy clay loam	Barely favourable	2
Torrecera	Cadiz	Spain	Luis Romero	**34**	-5.943	36.6091	Clay	Barely favourable	0
Utrera	Sevilla	Spain	Antonio Valle	**35**	-5.7792	37.1849	Clay	Barely favourable	0
Utrera	Sevilla	Spain	Lorenzo Calderon 1	**36**	-5.7792	37.1849	Sandy loam	Barely favourable	0
Utrera	Sevilla	Spain	Lorenzo Calderon 2	**37**	-5.7792	37.1849	Sandy	Barely favourable	0
Valdepeñas	Ciudad real	Spain	La Abundancia	**38**	-4.475	40.8361	Sandy clay loam	Barely favourable	0.31
Andújar	Jaen	Spain	Pedro Burgos M2 (Propio)	**39**	-4.0287	38.2055	Clay	favourable	0.4
El Carpio	Cordoba	Spain	Bartolome Carmona	**40**	-4.4968	37.941	Clay	favourable	0.4
Avileses	Murcia	Spain	J.M Guillamon (La Peraleja 2)	**41**	-0.9433	37.8495	Clay loam	favourable	6
Ecija	Sevilla	Spain	Pepe Ramirez 2 (Cortijo)	**42**	-5.0827	37.5415	Clay	favourable	12
Jerez de la Frontera	Cadiz	Spain	Juan Carlos Bernal	**43**	-6.1259	36.6853	Clay loam	favourable	0.4
Jerez de la Frontera	Cadiz	Spain	Jerez maiz M2	**44**	-6.1259	36.6853	Clay	favourable	10.8
Jerez de la Frontera	Cadiz	Spain	Jerez trigo M3	**45**	-6.1259	36.6853	Clay	favourable	8.8
Jerez de la Frontera	Cadiz	Spain	Jerez trigo M4	**46**	-6.1259	36.6853	Clay loam	favourable	0
Jerez de la Frontera	Cadiz	Spain	Jerez girasol M1	**47**	-6.1259	36.6853	Clay	favourable	1.2
Las Cabezas de San Juan	Seville	Spain	Enrique Gonzalez de la peña	**48**	-5.9382	36.9804	Sandy clay loam	favourable	0
Las Cabezas de San Juan	Sevilla	Spain	Antonio Valle 1 a 11	**49**	-5.9382	36.9804	Clay	favourable	0.4
Los Palacios	Seville	Spain	Juan Ramon Martinez M2	**50**	-5.9277	37.1631	Sandy clay loam	favourable	0

(Continued)

Supplementary Table S1. Continued.

City	District	Coutry	Field	Field number	Latit.	Longit.	Soil texture	Previous cropping history	Ms/g soil
Santaella	Cordoba	Spain	Finca Santa Margarita	**51**	-4.843	37.5622	Clay	favourable	0.4
Villarubia	Córdoba	Spain	Gerardo Amian (Algodón)	**52**	-4.9176	37.8484	Clay loam	favourable	0.8
Andújar	Jaen	Spain	Pedro Burgos M1 (Antiguo)	**53**	-4.0287	38.2055	Clay	very favourable	44.4
Ecija	Sevilla	Spain	Sotillo Gallego 1 (camino)	**54**	-5.0827	37.5415	Clay	very favourable	7.6
Ecija	Sevilla	Spain	Pepe Ramirez 3 (Cerca rio)	**55**	-5.0827	37.5415	Clay	very favourable	33.6
Ecija	Sevilla	Spain	Pepe Ramirez 1 (Cerca carretera)	**56**	-5.0827	37.5415	Clay	very favourable	12.4
Écija	Sevilla	Spain	Sotillo Gallego 2 (Transformador)	**57**	-5.0827	37.5415	Clay	very favourable	19.2
La Carlota	Cordoba	Spain	Federico Garcia M2	**58**	-4.9338	37.6726	Sandy clay loam	very favourable	4.8
Las Cabezas de San Juan	Seville	Spain	Enrique Gonzalez de la peña	**59**	-5.9382	36.9804	Clay	very favourable	20.4
Los Palacios	Seville	Spain	Juan Ramon Martinez M3	**60**	-5.9277	37.1631	Sandy loam	very favourable	0
Santaella	Cordoba	Spain	Federico Garcia M3	**61**	-4.843	37.5622	Clay	very favourable	1.6
Seville	Seville	Spain	La Corchuela 13	**62**	-5.9209	37.2829	Sandy loam	very favourable	0
Seville	Seville	Spain	La Corchuela 10	**63**	-5.9209	37.2829	Sandy loam	very favourable	0.8
Seville	Seville	Spain	La Corchuela 21	**64**	-5.9209	37.2829	Loam	very favourable	4
Almodovar	Cordoba	Spain	Galan (Pendiente)	**65**	-5.0146	37.8132	Clay loam	extremely favourable	8.4
Almodovar	Cordoba	Spain	Galan (gasolinera)	**66**	-5.0146	37.8132	Sandy clay loam	extremely favourable	46.6
Arjonilla	Jaen	Spain	Juan Carlos Roldan Zona 3	**67**	-4.1066	37.9745	Clay	extremely favourable	52
El Carpio	Cordoba	Spain	Santiago Estevez Palomar	**68**	-4.4968	37.941	Clay	extremely favourable	1.2
Espelúy	Jaen	Spain	Ecotex 1	**69**	-3.8623	38.0323	Loam	extremely favourable	12.26

(Continued)

Supplementary Table S1. Continued.

City	District	Coutry	Field	Field number	Latit.	Longit.	Soil texture	Previous cropping history	Ms/g soil
La Lantejuela	Sevilla	Spain	Rafael Calle	**70**	-5.2228	37.3544	Clay	extremely favourable	2.8
Las Cabezas de San Juan	Seville	Spain	Juan Bastos	**71**	-5.9382	36.9804	Sandy clay loam	extremely favourable	0
Seville	Seville	Spain	La Corchuela 6	**72**	-5.9209	37.2829	Clay loam	extremely favourable	1.2
Seville	Seville	Spain	La Corchuela 4	**73**	-5.9209	37.2829	Sandy clay loam	extremely favourable	10.4
Vejer de la Frontera	Cadiz	Spain	Finca Las Lomas P-42-4	**74**	-5.9667	36.252	Sandy clay loam	extremely favourable	0
Vejer de la Frontera	Cadiz	Spain	Finca Las Lomas P-42-2	**75**	-5.9667	36.252	Sandy clay loam	extremely favourable	0.4
Vejer de la Frontera	Cadiz	Spain	Finca Las Lomas P-42-3	**76**	-5.9667	36.252	Sandy clay loam	extremely favourable	0.8
Vejer de la Frontera	Cadiz	Spain	Finca Las Lomas P-42-5	**77**	-5.9667	36.252	Sandy clay loam	extremely favourable	0
Vejer de la Frontera	Cadiz	Spain	Finca Las Lomas P-42-1	**78**	-5.9667	36.252	Sandy clay loam	extremely favourable	0
Vejer de la Frontera	Cadiz	Spain	Finca Las Lomas P-50-1	**79**	-5.9667	36.252	Sandy clay loam	extremely favourable	6.8
Vejer de la Frontera	Cadiz	Spain	Finca Las Lomas P-50-2	**80**	-5.9667	36.252	Sandy clay loam	extremely favourable	24.4
Vejer de la Frontera	Cadiz	Spain	Finca Las Lomas P-50-3	**81**	-5.9667	36.252	Sandy loam	extremely favourable	10.4
Vejer de la Frontera	Cadiz	Spain	Finca Las Lomas P-50-4	**82**	-5.9667	36.252	Sandy clay loam	extremely favourable	15.6

(Continued)

Supplementary Table S1. Continued.

City	District	Coutry	Field	Field number	Latit.	Longit.	Soil texture	Previous cropping history	Ms/g soil
Vejer de la Frontera	Cadiz	Spain	Finca Las Lomas P-50-5	**83**	-5.9667	36.252	Sandy clay loam	extremely favourable	18.8
Villa del Río	Cordoba	Spain	Hnos. Caro	**84**	-4.2935	37.9814	Clay	extremely favourable	8.4

[1] long rotation had < 2 host crops to *V. dahliae* in five years; an intermediate rotation had host crops to *V. dahliae* 51-71% of the time; a none rotation indicated that all crops were susceptible to *V. dahliae*; a short rotation indicated that one crop in five years was not a host of *V. dahliae*.
